# Publisher Correction: Entanglement-enhanced matter-wave interferometry in a high-finesse cavity

**DOI:** 10.1038/s41586-022-05582-4

**Published:** 2022-12-06

**Authors:** Graham P. Greve, Chengyi Luo, Baochen Wu, James K. Thompson

**Affiliations:** JILA, NIST and Department of Physics, University of Colorado, Boulder, CO USA

**Keywords:** Matter waves and particle beams, Atomic and molecular interactions with photons, Quantum optics, Quantum simulation, Quantum metrology

Correction to: *Nature* 10.1038/s41586-022-05197-9 Published online 19 October 2022

In the version of this article initially published, for the *ħk* values shown in Fig. 1a,b and Fig. 2c–e, the modified constant *ħ* was omitted in error. In addition, the Peer review information mistakenly linked to an earlier version of the article manuscript and has since been removed. The changes are reflected in the HTML and PDF versions of the article.

